# Sustained Depolarization Induces Gene Expression Pattern Changes Related to Synaptic Plasticity in a Human Cholinergic Cellular Model

**DOI:** 10.1007/s12035-024-04262-w

**Published:** 2024-06-28

**Authors:** Anna Maria Carrese, Rossella Vitale, Manuela Turco, Valeria Masola, Francesco Aniello, Emilia Vitale, Aldo Donizetti

**Affiliations:** 1 Department of Biology, University of Naples Federico II, Naples, 80126 Italy; 2 Institute of Biochemistry and Cell Biology, National Research Council (CNR), Naples, 80131 Italy; 3 Department of Mental and Physical Health and Preventive Medicine, University of Campania “Luigi Vanvitelli”, Naples, 80138 Italy

**Keywords:** Synaptic Plasticity, Gene Expression, Cholinergic Neurons, SH-SY5Y Cells, Alzheimer's Disease

## Abstract

Neuronal gene expression in the brain dynamically responds to synaptic activity. The interplay among synaptic activity, gene expression, and synaptic plasticity has crucial implications for understanding the pathophysiology of diseases such as Alzheimer’s disease and epilepsy. These diseases are marked by synaptic dysfunction that affects the expression patterns of neuroprotective genes that are incompletely understood. In our study, we developed a cellular model of synaptic activity using human cholinergic neurons derived from SH-SY5Y cell differentiation. Depolarization induction modulates the expression of neurotrophic genes and synaptic markers, indicating a potential role in synaptic plasticity regulation. This hypothesis is further supported by the induction kinetics of various long non-coding RNAs, including primate-specific ones. Our experimental model showcases the utility of SH-SY5Y cells in elucidating the molecular mechanisms underlying synaptic plasticity in human cellular systems.

**Supplementary Information:**

The online version contains supplementary material available at 10.1007/s12035-024-04262-w.

## Introduction

At the core of optimal brain function lies synaptic activity, the intricate exchange of information between nerve cells. This activity triggers a cascade of responses within neurons, including the activation of activity-regulated genes (ARGs). These genes exert influence over crucial processes such as neuroprotection and synaptic plasticity, elements of the brain’s remarkable capacity to adapt and reconfigure neural connections. Synaptic connections undergo dynamic strengthening or weakening in response to fluctuations in neuronal activity. This phenomenon, known as synaptic plasticity, involves intricate processes such as the release and recycling of vesicles, trafficking of neurotransmitter receptors, mobilization of adhesion molecules, and alterations in gene expression within neurons [1]. These multifaceted mechanisms collectively influence the strength of synaptic connections, as highlighted by Cuestas Torres and Cardenas [2]. Furthermore, at the cellular level, activity-dependent synaptic plasticity is widely recognized as one of the fundamental mechanisms contributing to the processes of learning and memory  [3].

The central neurophysiological bases of memory are most likely activity-dependent changes in synaptic efficacy, such as long-term potentiation (LTP) and long-term depression (LTD) [4, 5]. These processes represent complementary mechanisms through which, depending on the pattern of activation of synaptic input, a long-lasting increase or decrease in synaptic efficacy may occur [6, 7].

Several central nervous system (CNS) disorders are characterized by synaptic dysfunction, which may be the cause or consequence of specific pathologies  [8, 9]. An example of synaptic dysfunction is found in Alzheimer’s disease (AD). AD is a multifactorial disorder in which aberrant enhancement of excitatory activity is one of the earliest changes observed in cortical and hippocampal circuits in AD patients and model mice [10]. This enhanced activity occurs early, before the onset of clinical symptoms, and is associated with the progression of cognitive impairment [11] and changes in synaptic plasticity [12]. These changes include the impairment of long-term potentiation (LTP) and the facilitation of long-term depression (LTD) [13–17].

Synaptic malfunctions also underlie other types of diseases, such as epilepsy, which is characterized by the occurrence of spontaneous recurrent seizures (SRS) generated by an imbalance of excitatory and inhibitory synaptic transmissions that induce abnormally synchronized electrical activity [18–22]. This process is characterized by progressive cellular and molecular changes that lead to the reorganization of the neuronal network. Alterations in long-term potentiation (LTP), like those observed in human epileptic tissue, can be reproduced in animal models of temporal lobe epilepsy (TLE), the most common form of epilepsy, through the administration of chemoconvulsants [23–26].

A particular aspect of synaptic plasticity that has rarely been explored is the role of non-coding RNAs (ncRNAs), which are particularly abundant in the central nervous system. Alterations in their expression patterns have been linked to neuronal differentiation and function, as well as long-term memory formation [27].

Among these ncRNAs, the best known are microRNAs (miRNAs), but increasing attention is being paid to long non-coding RNAs (lncRNAs), which serve as key regulators of gene expression in neurons. Their mechanism of action is based on their ability to interact with other molecules, facilitated by their structural plasticity. Depending on their localization and specific interactions (with DNA, RNA, or proteins), they can act at different stages of gene expression and participate in processes ranging from chromatin remodeling to transcriptional, post-transcriptional, and epigenetic regulation [28, 29]).

An imbalance in lncRNA expression is associated with several diseases, including cancer  [30] and neurodegenerative diseases [31–34]. For example, several studies evaluating the profiles of abnormally expressed transcripts in animal models of AD showed that most of the lncRNAs upregulated or downregulated in AD were linked to metabolic pathways, inflammatory processes, and even synaptic transmission [34–36].

Expression of lncRNAs has also been shown in the literature to be regulated in processes such as synaptogenesis and in response to various stimuli, including brain-derived neurotrophic factor (BDNF), which is critical for neural survival and plasticity [37]. They respond to synaptic activity [38], with some exhibiting kinetics similar to immediate early genes (IEGs) [39].

In recent years, a large amount of data has accumulated on the study of synaptic plasticity using various available models, such as in vitro, ex vivo, and in vivo, along with different electrophysiological approaches [40].

However, understanding the initiation and contribution of synaptic dysfunction in neurological disorders is difficult because of limited access to human tissue samples and because of possible differences in pathological mechanisms between existing experimental animal models and humans. This is especially the case in the study of disorders affecting the brain, where the most significant differences are found [41].

Cell cultures, and in particular human cell cultures, offer a viable alternative. They serve as the only reproducible, ethical, and versatile human model system. With their ability to study disease mechanisms in a human cellular context, they are particularly useful. Additionally, they facilitate the study of neuronal excitation-transcription coupling [42, 43].

Within this spectrum, the SH-SY5Y cell line stands out as one of the most extensively employed in neurobiology. Its neuroblastic nature facilitates cost-effective and straightforward propagation, while its capacity to differentiate under the influence of morphogens enables the development of terminally mature neurons [44].

In this paper, we present a human mature neuronal model, in which synaptic plasticity in response to a pattern of synaptic activity can be studied in vitro to gain an understanding of neurological diseases.

## Methods

### Cell Culture

N-enriched SH-SY5Y (human neuroblastoma, ATCC®, Manassas, VA, USA) cell line was grown and propagated in Dulbecco’s Modified Eagle’s Medium (DMEM, EuroClone®, Milan, Italy) supplemented with 2 mM L-glutamine (EuroClone®, Milan, Italy), a solution of 1% penicillin/streptomycin (EuroClone®, Milan, Italy), and 15% fetal bovine serum (FBS, EuroClone®, Milan, Italy). In particular, the N-enriched population of SH-SY5Y was obtained from the parental cell line by a procedure reported elsewhere [37]. Cells were cultured and maintained in a 5% CO_2_ humidified incubator at 37 °C.

### Differentiation of Cell Cultures

The N-enriched SH-SY5Y cells were differentiated by incubation in a low serum (1.5%) medium containing 10 µM retinoic acid (ATRA) (RA, SIGMA-Aldrich®, St. Louis, MO, USA) and 50 ng/mL BDNF (PeproTech®, London, UK). In particular, 8 × 10^5^ cells were seeded in 35-mm plates and stimulated by the differentiation medium, which was refreshed every 2 days. The differentiation process was monitored by LEICA DMi8 microscope and images were taken at 0 and 12 days of differentiation.

### Morphometric Analysis

Image processing and analysis were performed by Fiji software (ImageJ). The NeuronJ plug-in was used to quantify the number and length of neurites [45]. Primary and secondary neurites were plotted semiautomatically using 9 images per experimental condition; then the number and total length of neurites (in inches) were normalized to the number of neurons to obtain the number of neurites per neuron in each image. Finally, to analyze the branching of neurites, the nodes (the points of the primary neurites from which the secondary neurites branch) and the total number of cells were counted for each image by using a preformed grid, obtaining the number of nodes per cell.

### Depolarization Protocol

A solution with high concentrations of potassium (Depolarization solution) (Table S1, Supplementary Materials) was used to in vitro stimulate this process. Depolarization was performed by adding Depolarization solution to a final concentration of 31% directly into the neuronal culture medium to reach 55mM KCl and incubated for 1’, 1 h, and 8 h. Cell viability was evaluated by visual inspection of the cell morphology. No evident changes in the morphology and number of the cells were revealed.

### Synaptic Vesicles Recycling

Cultures were stained with AM1-43 styryl dye (Biotium, Hayward, CA, USA). Specifically, the cells were incubated with 4 µmol/L AM1-43 in the depolarization solution. Cells were washed in cold PBS, were fixed for 20 min with 4% paraformaldehyde, and then washed in cold PBS. The cells were observed by a JuliStage fluorescent microscope. Specific laser parameters used for the analysis are reported in Supplementary Materials (Table S2). The data were obtained from 1 field from each of 3 independent biological replicates.

The level of synaptic vesicle recycling was verified by measuring the number of fluorescent puncta in untreated cells (CTRL) and depolarized cells after 1’ and 1 h. Each fluorescence image was divided into 9 quadrants that were further magnified. The sizes of the fluorescence puncta were evaluated by the support of Nucleus Counter plugin of Image J in order to exclude puncta larger than 40 pixels. Only smaller puncta were considered as vesicles and manually counted by visual inspection and confirmed by the analysis. The obtained counts of fluorescence vesicles were divided by the number of cells obtained by manually counting from the corresponding bright field images.

### RNA Isolation, Retrotranscription and Quantitative PCR Analysis

Total cellular RNA was isolated using TRItidy G (AppliChem®, Germany) according to the manufacturer’s instructions. The concentration and purity of the RNA samples were assessed using a NanoDrop® 1000 spectrophotometer (Thermo Fisher, Waltham, MA, USA). 1 µg of RNA was reverse transcribed into cDNA using Luna Script RT SuperMix® (New England Biolabs, Ipswich, MA, USA). qPCR was performed on three independent biological replicates, in technical duplicate for each biological replicate using the SYBR green (SYBR® Green GDSBio, Guangpu East Road, Huangpu District, Guangzhou, Guangdong, China) method. The reaction mixture contained 20 ng of cDNA template and 400 nM of each forward and reverse primer in a final volume of 15 µL. The PCR conditions included a denaturation step (95 °C for 10 min) followed by 40 cycles of amplification and quantification (95 °C for 35 s, 60 °C for 1 min). The relative gene expression levels were normalized to the reference gene Hypoxanthine Phosphoribosyltransferase 1 (*HPRT1*) and calculated by the 2^−ΔΔCt^ method. The data are reported in the graph as Log_2_FC. The sequences of the primers are listed in Supplementary Materials, Table 3.

### Statistical Analysis

The results from independent biological replicates are expressed as mean ± SEM. Statistical analysis of the data was carried out using a two-tailed t-test and ANOVA test (GraphPad Prism Software, San Diego, CA, USA) with a p-value cut-off of 0.05.

## Results

### SH-SY5Y Differentiation

**Fig. 1:**
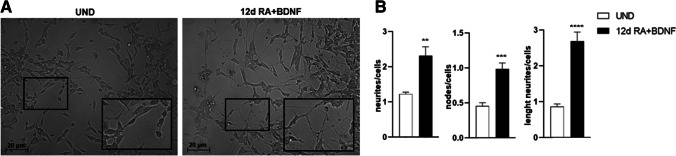
Morphological comparison between phase-contrast images of undifferentiated and neuron-like cells at 12 days differentiation RA + BDNF-induced. Undifferentiated SH-SY5Y (UND) displayed short processes, while differentiated SH-SY5Y (12d RA + BDNF) showed branched and elongated neurites (**A**); graphs of morphometric parameters of undifferentiated and differentiated cells (**B**); Values are reported as Mean ± SEM. Statistical analysis of the data was carried out using a two-tailed t-test. **: *p* ≤ 0.01, ***: *p* ≤ 0.001, ****: *p* ≤ 0.0001

Synaptic activity requires the formation of functional synapses downstream of the neuron maturation process. For the present work, a combination of all-trans retinoic acid and BDNF in a culture medium with reduced FBS concentration was used to obtain mature neurons. The maturation process was carried out for 12 days and was monitored by light microscope observations. As shown in Fig. 1, the maturation process is evident from the change in the morphology of the soma, which passes from roundish into pyramidal, and from the elongation and arborization of the neurites to form a dense network (Fig. 1A).

The number of nodes, the length, and the number of neurites in SH-SY5Y cells were measured on day 0 and day 12, where a significant increase in all the analyzed morphometric parameters was observed (Fig. 1B).

**Fig. 2:**
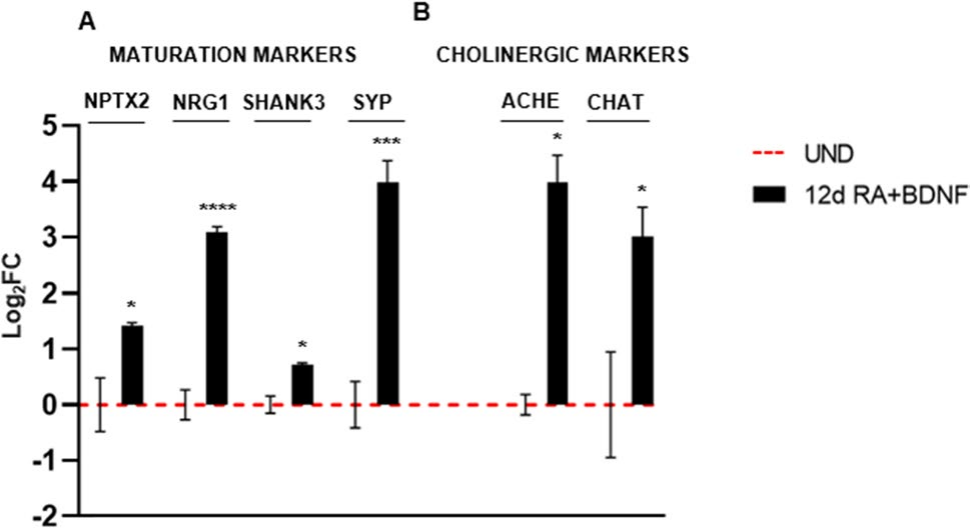
Gene expression of synaptic markers in differentiated cells (12d RA + BDNF) compared to undifferentiated cells (UND) (**A**); neuronal subtype markers expression at 12 days of differentiation (**B**). The gene expression level was normalized against the reference *HPRT1* transcript and calculated as Log_2_FC. Values are reported as Mean ± SEM. Statistical analysis of the data was carried out using a two-tailed t-test. *: *p* ≤ 0.05, ***: *p* ≤ 0.001, ****: *p* ≤ 0.0001

Robust differentiation was also confirmed by gene expression analysis of synaptic proteins, such as NPTX2, NRG1, SHANK3, and SYP, which were found to be upregulated in SH-SY5Y cells at day 12 of differentiation compared with undifferentiated SH-SY5Y cells (Fig. 2A).

The neuronal subtype of SH-SY5Y-derived neurons depends on the differentiation procedure [44]. The combined treatment of RA and BDNF promotes differentiation towards the cholinergic subtype [46]. We confirmed this effect by the analysis of the gene expression level of two cholinergic markers (*ACHE* and *CHAT*) (Fig. 2B).

### Induction of Depolarization

Following the characterization of the neuronal subtype, we aimed to induce robust synaptic activity in this cellular model by stimulating depolarization. Synaptic activity consists of the release of neurotransmitters, contained in synaptic vesicles, into the synaptic cleft upon arrival of the nerve impulse. Exocytosis of vesicles can be artificially stimulated through a series of treatments with chemical agents or more simply through high extracellular concentrations of KCl that induce a process of membrane depolarization, simulating synaptic activity. Different patterns of neuronal activation could be coupled to a different gene expression profile when cells are stimulated by brief or sustained depolarization by a high-potassium solution [43]. In this regard, we wanted to compare two different treatment times (1’ and 1 h) to induce depolarization by a final KCl concentration of 55 mM.

**Fig. 3:**
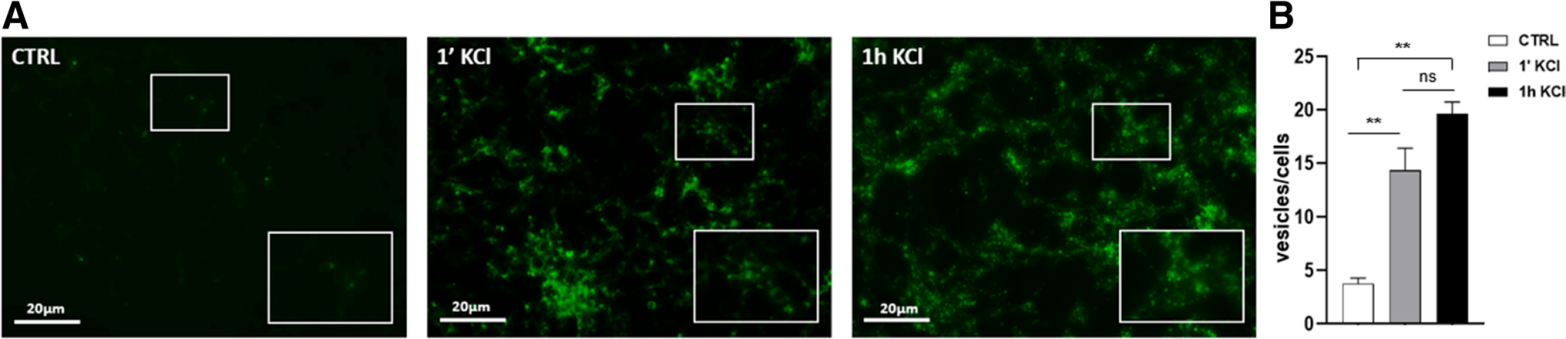
Microscopy fluorescent images, in which synaptic vesicles are visible in green (**A**); quantitative analysis of vesicles in differentiated cells treated with Depolarization solution, at two different times (1’ and 1 h), and unstimulated cells (CTRL) (**B**). Statistical analysis of the data was carried out using a one-way ANOVA test. **: *p* ≤ 0.01; ns: not significant

We evaluated synaptic activity by fluorescent labeling of vesicles, as reported in Materials and Methods. The control (CTRL), consisting of unstimulated cells, has a very low number of vesicles, which are found to increase already after 1’ of treatment, and even more after 1 h of treatment, where the highest number of vesicles are observed (Fig. 3).

**Fig. 4:**
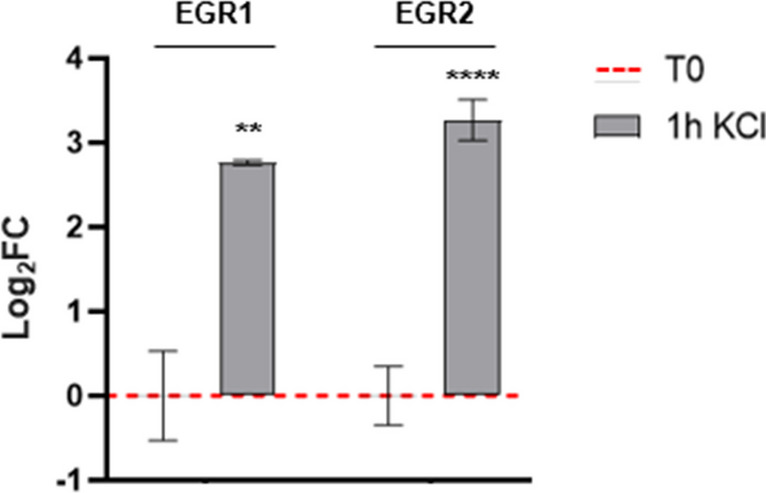
Expression levels of IEG *EGR1* and *EGR2* following depolarization in cells stimulated for 1 h. The gene expression level was normalized against the reference *HPRT1* transcript and calculated as Log_2_FC. Values are reported as mean ± SEM. Statistical analysis of the qPCR data was carried out using a two-tailed t-test. **: *p* ≤ 0.01; ****: *p* ≤ 0.0001

To further validate the efficacy of neuronal depolarization, we analyzed the induction of activity-regulated gene expression, a class of genes that respond to synaptic activity. We performed the expression analysis after sustained stimulation (1 h) with KCl, considering that continuous treatment ensures robust rapid and delayed gene expression [43]. The depolarization protocol leads to a significant increase of the analyzed immediate early genes after 1 h of treatment, corroborating the efficacy of the treatment (Fig. 4).

### Kinetics of Primary and Secondary Response Genes Expression

To understand the impact of sustained stimulation on synaptic plasticity, we sought to examine the expression kinetics of genes associated with neuronal health. This includes synaptic markers such as *SHANK3*, *SYP*, and *NPTX2*, along with pro-survival genes like *BDNF*, *NTRK2*, *PGRN*, and *NR4A1*. To delve into the temporal aspects of gene induction, we analyzed their expression levels at both 1 h and 8 h, enabling an exploration of both primary and secondary response gene expression patterns.

**Fig. 5:**
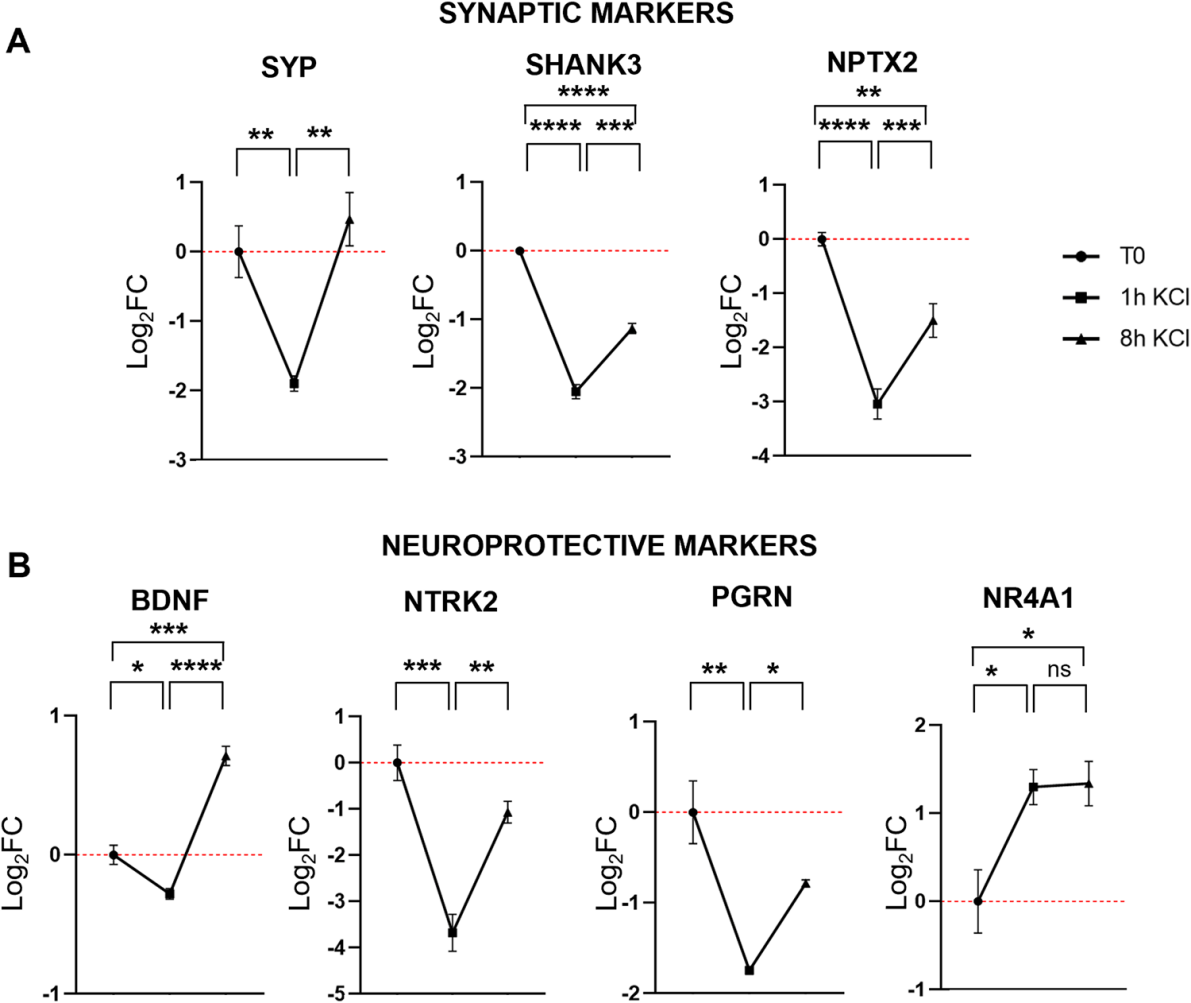
Expression levels of synaptic markers (**A**) and neuroprotective markers (**B**) following depolarization in cells treated for 1 h and 8 h. The gene expression level was normalized against the reference *HPRT1* transcript and calculated as Log_2_FC. Values are reported as mean ± SEM. Statistical analysis of the qPCR data was carried out using a one-way ANOVA test. *: *p* ≤ 0.05; **: *p* ≤ 0.01; ***: *p* ≤ 0.001; ****: *p* ≤ 0.0001; ns: not significant

Real-time PCR analysis of gene expression revealed a notable reduction (approximately 2-3-fold), in the expression levels of *SHANK3*, *SYP*, and *NPTX2* after 1 h of treatment in comparison to non-stimulated controls. Subsequently, these expression levels exhibited a marked and significant increase at the 8 h time point compared to the expression level at 1 h (Fig. 5A).

A comparable pattern is evident in genes associated with neuroprotection, including *BDNF*, *NTRK2*, and *PGRN*. Notably, *NR4A1* exhibits an early increase in expression levels as early as 1 h post-treatment, persisting in expression even after the 8 h time point (Fig. 5B).

### Expression Kinetics of Long Non-coding RNAs

**Fig. 6:**
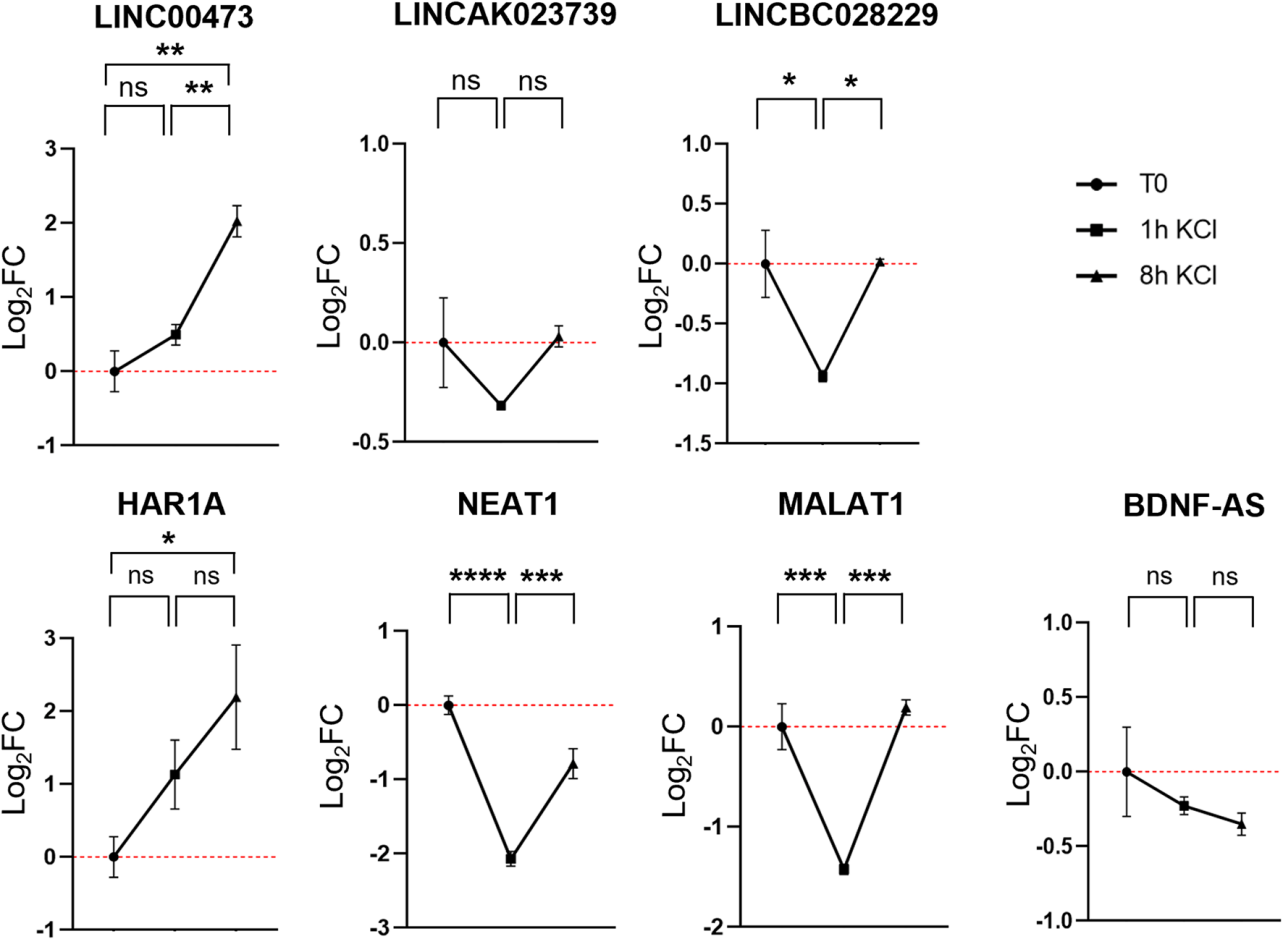
Expression levels of long non-coding RNA following depolarization in cells treated for 1 h and 8 h. The gene expression level was normalized against the reference *HPRT1* transcript and calculated as Log_2_FC. Values are reported as mean ± SEM. Statistical analysis of the qPCR data was carried out using a one-way ANOVA test. *: *p* ≤ 0.05; **: *p* ≤ 0.01; ***: *p* ≤ 0.001; ****: *p* ≤ 0.0001; ns: not significant

Given the pivotal role that long non-coding RNAs (lncRNAs) play in orchestrating various facets of development, homeostasis, and plasticity within the nervous system, we aimed to delineate their expression profiles in our experimental paradigm. Specifically, as illustrated in Fig. 6, the induction of synaptic activity stimulated the expression of the primate-specific lncRNA *LINC00473* and* HAR1A*, as evident after 8 h of stimulation. In contrast, KCl treatment led to a significant reduction in the levels of *NEAT1*, *MALAT1*, and *LINCBC028229* after 1 h of treatment, which then increased significantly after 8 h (Fig. 6) On the other hand, the transcript level of *LINCAK023739* and *BDNF-AS*, did not appear to vary at the treatment times analyzed (Fig. 6).

## Discussion

The neuroblastoma cell line SH-SY5Y is widely employed in neurobiology due to its numerous advantages over alternative cellular models, notably its capacity to differentiate into various adult neuronal subtypes. Undifferentiated SH-SY5Y cells exhibit markers associated with immature neurons as well as glial cells and their progenitors [47, 48]. Consequently, differentiation is imperative to characterize these cells as mature neurons suitable for specific experimental inquiries, such as the investigation of neurological diseases impacting several neuronal subtypes.

In the context of our study, the applied differentiation protocol, using a combination of RA and BDNF over 12 days, yielded a cholinergic neuronal model particularly useful for Alzheimer’s disease (AD). The examination of synaptic activity is achievable in vitro, offering insight into the intricate interplay between neuronal excitation and gene expression. Synaptic activity, accompanied by the subsequent influx of Ca^2+^ serves as a pivotal mechanism facilitating communication between the synapse and the nucleus. This process activates second messengers and initiates gene transcription, playing a crucial role in neural circuit function, from synapse formation during developmental stages to synaptic plasticity in the mature brain [49].

This experimental model can therefore be fundamental for the study of neurodegenerative diseases, many of which are characterized by synaptic dysfunction. For example, the early stages of Alzheimer’s disease are marked by a dysfunction in hippocampal synaptic activity. This impairment contributes to a deficiency in hippocampal-dependent memory, reliant on activity-dependent modifications in synaptic efficacy. These alterations, in turn, have the potential to either facilitate or disrupt rhythmic electrical activity, underscoring a bidirectional relationship  [40]. To study this correlation and to refine the characterization of our experimental model for in vitro synaptic activity studies, we induced sustained depolarization. In particular, to analyze downstream activated genes, we used a final KCl concentration of 55 mM, that is considered to induce full-strength depolarization in primary neurons, and gene induction, like synaptic stimulation  [50]. A prolonged treatment with 55 mM of KCl was also successfully used to induced activity-dependent gene expression in human fetal brain cultures and GABAergic neurons differentiated from iPSCs  [51, 52].

In neurons, depolarization leads to the expression of transcription factors (TFs), and expression analysis of *EGR1* and *EGR2*, at time 1 h, validated our depolarization model. In addition, we also sought to analyze the kinetics of another TF, *NR4A1*, which is found to increase at both time points (1 h and 8 h) of our experimental paradigm, in agreement with data already obtained on mouse cortical neurons  [43].

We also analyzed the gene expression levels of the neurotrophic factor BDNF and its receptor NTRK2 because generally, neuronal activity regulates the expression, processing, transport, and release of neurotrophic factors, many of which have well-characterized neuroprotective effects [8, 53]. The expression of BDNF increased between 1 h and 8 h of depolarization in a significant manner also in the comparison between unstimulated and 8 h after KCl stimulation, in agreement with its behavior as a Late-Response Gene [43, 51, 52]. In contrast, NTRK2 showed a significant increase only between 1 h and 8 h. This pattern was also evident for PGRN, another neurotrophic factor examined, which is highly colocalized and co-transported with BDNF, and, similarly, is recruited to synapses and secreted in an activity-dependent manner [54].

Because at the cellular level synaptic plasticity is mediated by structural changes (elongation, contraction, and shape changes) of synapses [55], we analyzed the expression levels of several synaptic markers, including *SYP*, *SHANK3*, and *NPTX2*. Synaptophysin, is an integral membrane protein localized to synaptic vesicles, involved in synaptic function including exocytosis, synapse formation, biogenesis, and synaptic vesicles endocytosis [56–58], SHANK3 is a central scaffold protein for postsynaptic densities (PSDs) that promotes the development of dendritic spines [59, 60], and NPTX2 is a secreted synaptic protein considered as a marker of structural and functional synaptic deficits in neurodegeneration [61].

The expression patter of *NPTX2* here reported is differs from that of [43]. This is not surprising, considering that human and mouse synaptic activity-induced transcriptional programs share many genes but at the same time, genetic differences, for instance primate-specific long non-coding RNA, account for lineage-specific gene expression kinetics that can have an impact on synaptic activity-dependent transcription [38]. In addition, differences in gene expression pattern after KCl-induced depolarization can be cell types-specific and strictly related to the category of genes, with ERGs (Early-Response Genes) consistently induced across different cell types [52]. In fact, in our analysis, the induction of ERG encoding for nuclear protein, in particular transcription factors, is confirmed, while differences are found for the secondary-response genes. Our analysis has been focused on two time points, and thus cannot exhaustively depict the complex expression kinetics of different genes. Nevertheless, our data demonstrate that, for the analyzed late-response genes following an RNA decay process at an early time after KCl stimulation (1 h), new transcription is evidenced by increased transcript levels at 8 h compared to 1 h, likely due to the induction of transcription factor encoded by the primary responsive genes (also known as immediate early genes). We interpreted this new transcription as evidence of induction of those mechanisms involved in synaptic plasticity. This suggests synaptic reorganization, since synapse formation and stabilization in the nervous system is a dynamic process [62]. Further analyses need to be conducted to gain a more comprehensive understanding of the effects of depolarization induction on synaptic plasticity, including protein expression. The expression of *BDNF* is generally regulated by its natural antisense transcript (NATs) *BDNF-AS*  [63], but our analyses show no change in its levels, which might reinforce the idea that the observed kinetics for *BDNF* is not due to the effect of this NAT, but to synaptic remodeling. BDNF regulates several biological functions implicated in neuronal survival, differentiation, and synaptic plasticity, participating in both the early stages of LTP and LTD [7, 64, 65]. At the same time, synaptic activity regulates synaptic structure by precisely regulating the synthesis of BDNF  [66]. The analysis of BDNF and PGRN gene expression in the present work likely is a consequence of a dynamic process where *BDNF* and *PGRN* mRNAs, which generally localized in distal dendrites [67], could be regulated in response to our treatment. Furthermore, to assess a time course analysis of the induction of long non-coding RNAs downstream of synaptic activity, the expression of *LINC00473*, a primate-specific lncRNA regulated by synaptic activity, is analyzed. It is hypothesized to control the kinetics of gene expression in the immediate early response [38, 39] and was found to increase as early as 1 h of treatment, and then further increased significantly at time 8 h, as similarly reported in Boulting et al. [52] and Ataman et al. [51], confirming the validity of our experimental paradigm in the study of lncRNAs.

Long non-coding RNAs can regulate several neuronal functions. We elected to investigate lncRNAs involved in the regulation (in the formation and/or maintenance) of synapse density and dendritic arborization in neurons, such as *MALAT1*, which demonstrated the same trend as previously analyzed synaptic markers.

This agrees with previous observations in which knockdown/overexpression of *MALAT1* in cultured neuronal cells results in decreased/increased synaptic density  [68], supporting the idea of possible synaptic reorganization.

Brain-specific lncRNA sequences do not show high evolutionary conservation. Among them, *HAR1A* is characterized by a *HAR* portion (of 118 bp), in which there are about 18 mutations, compared to the *HAR* sequence of chimpanzees. It is thought to be critical in neurodevelopmental processes such as synapse development [69]: in our experimental model, its levels increase, at time 8 h, further underscoring the importance of a human neuronal model such as SH-SY5Y for investigating the human brain.

However, we found different induction kinetic in two other primate-specific lncRNAs dependent on electrical activity, *LINCAK023739* and *LINCBC028229*, and for *NEAT1*, associated with epilepsy. The latter two decrease at time 1 h, and then increase at time 8 h in a statistically significant manner.

*LINCAK023739* and *LINCBC028229* are directly regulated by MAPK signaling, which is critical for dendritic spine stabilization and long-term potentiation [70–72], likely contributing to the development and maintenance of epileptic activity; *NEAT1*, on the other hand, is dynamically down-regulated in response to neuronal activity in vitro and in vivo, but in addition, its dysregulation has been shown to render neurons susceptible to seizure activity in vivo [73]. Overall, our data suggest that the experimental model here presented is suitable for analyzing activity-regulated genes involved in synaptic plasticity.

## Conclusion

We developed an SH-SY5Y-based model of synaptic activity that shows synaptic plasticity. This cellular model is differentiated toward a cholinergic subtype and can be used to study the relationship between activity-regulated coding and genes in brain pathologies including Alzheimer’s disease.

## Electronic Supplementary Material

Below is the link to the electronic supplementary material.

Supplementary Material 1

## Data Availability

No datasets were generated or analysed during the current study.

## Abbreviations

ARGs: Activity-regulated genes
LTD: Long Term Depression
LTP: Long Term Potentiation
CNS: Central nervous system
AD: Alzheimer’s disease
SRS: Spontaneous recurrent seizures
TLE: Temporal lobe epilepsy
ncRNAs: Non-coding RNAs
miRNAs: MicroRNAs
lncRNAs: Long non-coding RNAs
IEGs: Immediate early genes
DMEM: Dulbecco’s Modified Eagle’s Medium
FBS: Fetal Bovine Serum
RA: Retinoic acid
BDNF: Brain-derived neurotrophic factor
HPRT1: Hypoxanthine Phosphoribosyltransferase 1
NPTX2: Neuronal Pentraxin 2
NRG1: Neuregulin 1
SHANK3: SH3 And Multiple Ankyrin Repeat Domains 3
SYP: Synaptophysin
ACHE: Acetylcholinesterase
CHAT: Choline O-Acetyltransferase
EGR1: Early growth response protein 1
EGR2: Early Growth Response 2
NTRK2: Neurotrophic Receptor Tyrosine Kinase 2
PGRN: Progranulin
NR4A1: Nuclear Receptor Subfamily 4 Group A Member 1
NEAT1: Nuclear Enriched Abundant Transcript 1
HAR1A: Highly Accelerated Region 1 A
BDNF-AS: BDNF Antisense RNA
TF: Transcription factor
PSDs: Postsynaptic Densities
ERGs: Early-Response Genes
NAT: Natural antisense transcript
