# Supplementary material for: Sustained Depolarization Induces Gene Expression Pattern Changes Related to Synaptic Plasticity in a Human Cholinergic Cellular Model
Source: Mol Neurobiol. 2024 Jun 28;62(1):935–45. doi: 10.1007/s12035-024-04262-w (PMC11711863; doi:10.1007/s12035-024-04262-w)
Supplement: Supplementary file 1 — Supplementary Material 1 [file 12035_2024_4262_MOESM1_ESM.pdf]

Supplementary Materials

	$[X_f]$
KCl	170 mM
$MgCl_2$	1 mM
Hepes (pH7.4)	10 mM
$CaCl_2$	2 mM
H_2O	

Table S1. Recipe Depolarization Solution.

	FITC
Laser	10.0
Gain	615

Table S2. JuliStage parameters.

Gene symbol	Sequence	Accession
ACHE	F: CTCAGCGCCACCGACAC<br>R: CTGGTTCTTCCAGTGCACCA	NM_001367919.2
BDNF	F: ACACAAAAGAAGGCTGCAGG<br>R: TGCTATCCATGGTAAGGGCC	NM_170734.4
BDNF-AS	F: GTGGGTCCATTCCGTGTGTG<br>R: AGCTGGTGCAGGTATCAGATTAG	NR_002832
CHAT	F: CGAGGAGAGCAGGTCCACA<br>R: TTTGCTGCCATCTTACGGGG	NM_001142929.2
EGR1	F: GAGCAGCCCTACGAGCAC<br>R: GAGTGGTTTGGCTGGGGTAA	NM_001964.3
EGR2	F: TCGGTGACCATCTTTCCCAA<br>R: GTTGATCATGCCATCTCCGG	NM_000399.5
HAR1A	F: AGCGGCGGAAATGGTTTCTA<br>R: CATCGCGGAAAACGGGATTC	NR_003244.2
HPRT 1	F: TATGCTGAGGATTTGGAAAGGGT<br>R: CGAGCAAGACGTTCAGTCCT	NM_000194.3
LINC00473	F: AAACGCGAACGTGAGCCCCG<br>R: CGCCATGCTCTGGCGCAGTT	NR_026860.1
***LINC* AK023739**	F: AACCACTTCCTCCTCTGCAC<br>R: TCTGGTTGTCTCCGAACACA	NR_110225.1
***LINC* BC028229**	F: TCCTGATAGACGTTCCCAAGG<br>R: CTAGCAGCAGGTTTCGATGG	NR_149073.1
MALAT1	F: TGGATGTGTAACTGAGGCGG<br>R: ACACCTTGAGTCATTTGCCT	NR_002819.4
NEAT1	F: ACAGCATTCCTGTCTGCGAA<br>R: GACTTCAGGCTCCAGCCATT	NR_028272.1
NPTX2	F: TGGACAAGAGCAGGACACCG<br>R: GGTCCCATATGTTGAACTGGCT	NM_002523.3
NRG1	F: TGTGCGGAGAAGGAGAAAAC<br>R: GGCAGCGATCACCAGTAAAC	NM_013962.3
NTRK2	F: CGGGGACTTTGGGATGTC<br>R: CGCTTTCCGTCGTGAATTTC	NM_006180.6

PGRN	F: AGCTGGGTGGCCTTAACAG<br>R: CAGTGGGCATCAACCTGG	NM_002087.4
SHANK3	F: CTGCCCTACCTGGAGTTTCG<br>R: TCAGGTTCGCCTTTGTGTGA	NM_001372044.2
SYP	F: ACTATGGGCAGCAAGGCTAC<br>R: CTGGGCTTCACTGACCAGAC	NM_003179.3

Table S3. Validated primer sequences.
